# Foot Pathologies in Dairy Cows: Preliminary Data on Clinical Assessment, Blood Biochemistry Analysis, and Infrared Thermography

**DOI:** 10.3390/ani16050790

**Published:** 2026-03-03

**Authors:** Filippo Spadola, Nicolò Parisi, Andrea Spadaro, Esterina Fazio, Enrico Fiore, Giorgia Taio, Giuseppe Piccione, Francesca Arfuso, Maria Rizzo

**Affiliations:** 1 Department of Veterinary Sciences, University of Messina, Polo Annunziata, 98168 Messina, Italy; filippo.spadola@unime.it (F.S.); nicolo.parisi@studenti.unime.it (N.P.); andrea.spadaro@unime.it (A.S.); gpiccione@unime.it (G.P.); farfuso@unime.it (F.A.); rizzom@unime.it (M.R.); 2 Department of Animal Medicine, Productions and Health (MAPS), University of Padua, 35020 Legnaro, Italy; enrico.fiore@unipd.it (E.F.); giorgia.taio@phd.unipd.it (G.T.)

**Keywords:** lameness, dairy cows, infrared thermography, foot disease

## Abstract

Foot diseases represent a major cause of impaired welfare and reduced productivity in dairy ruminants. This pilot study aimed to evaluate the clinical progression of foot lesions in dairy cattle by combining clinical examination with hematochemical analyses and infrared thermography of foot (central zone, R1; interdigital area, R2; lateral claw, R3; medial claw, R4), in order to assess the effectiveness of therapeutic interventions and the evolution of inflammation over time. The study involved dairy cows affected by different foot disorders that underwent functional and therapeutic trimming. Ten lactating cows with hoof diseases (DG) were tested and 5 healthy animals (CG) were selected to serve as a control group. The animals were evaluated and sampled (blood sampling and acquisition of thermographic foot images) at three different time points: clinical diagnosis of the foot pathology (T0), 24 h (T1) and 10 days (T2) after treatment. Higher neutrophils, lymphocytes, platelets, α1-(*p* < 0.05) and α2-fraction values were found in diseased subjects compared to healthy ones at T0 (*p* < 0.05). Thermography highlighted significantly higher foot thermal values in animals with lesions than in healthy animals (*p* < 0.01), especially at T0. Animals affected by hoof diseases showed lower foot temperature values at T1 compared to T0 and T2 (*p* < 0.001). Though the small sample size and the short observation period limited the possibility of extending the results to the entire cow population, the infrared thermography proved to be a valuable non-invasive tool for detecting active lesions and monitoring healing, with higher temperatures corresponding to inflamed areas and a progressive normalization after treatment. The combined evaluation of clinical signs, systemic inflammatory markers and thermographic patterns provided an integrated perspective on the progression of foot diseases.

## Simple Summary

The aim of this pilot study was to evaluate the clinical progression of foot lesions in dairy cows using a multidisciplinary approach, including clinical examination, hematochemical analyses, and infrared thermography (IRT). The results showed that cows affected by foot pathology had higher concentrations of immune and inflammatory indices and higher thermal values in the foot than healthy cows. The integration of IRT and acute phase protein assessment into routine monitoring may improve early diagnosis, enable timely interventions, and ultimately enhance animal welfare and the sustainability of dairy herds.

## 1. Introduction

The claw diseases and the lameness are major welfare problems in dairy cattle, causing pain, discomfort, and limitations in normal behavioral activities [1].

Lameness, one of the main causes of production losses, second only to mastitis, represents a significant problem for the economics of dairy farms as well. Studies in dairy cows show considerable variability in lameness prevalence indicating that some herds manage to maintain good control of the problem while others exhibit particularly high levels of lameness, making it difficult to precisely quantify its impact [1,2,3]. Economic losses associated with lameness represent a significant portion of dairy farm management costs. Within the European Union, these costs show great variability, influenced primarily by the effectiveness of prevention programs adopted on different farms. Specifically, the economic impact of lameness is reflected in three main production parameters: milk losses constitute the largest share, with an average reduction estimated at around 40%; fertility disorders follow, accounting for approximately 30% of costs; and therapeutic treatment costs contribute a further 30% [4]. Milk production declines even before treatment begins, and the extent of the loss depends on the type of lesion present, worsening further with late intervention. High-producing cows are more prone to lameness, which is why production and quality losses (milk protein and fat) are often particularly severe in highly efficient herds. These alterations impact farm profitability and can also have repercussions on milk quality, food supply chains, and food safety. Lameness also significantly compromises reproductive efficiency: in cows that show clinical lameness within the first 70 days of lactation, pregnancy rates are reduced by up to 25% compared to healthy animals [1,2,3,4,5,6]. Irregular cycles, anestrus, and an increased incidence of ovarian cysts have also been reported, while systemic inflammation associated with foot lesions appears to contribute to the prolonged interval to conception. Even in the early stages of the disease, before and during the breeding season, reproductive efficiency is significantly lower than in non-lame animals. From a health perspective, lameness is associated with longer lying times, increasing the risk of mastitis and intra-mammary infections. From a welfare perspective, although it is difficult to quantify the emotional impact of chronic pain, its negative effect cannot be underestimated. Finally, lameness contributes to early culling and lower carcass weight, resulting in a reduction in the final economic value. Because lameness is also linked to other farm issues—such as infertility, reduced production, and premature culling—the costs of these secondary conditions should be included in the overall economic evaluation to more accurately represent the true impact of lameness within the herd [3]. One reason for such high losses is the slow progression of the disease: lameness often progresses for weeks or months before being diagnosed and can persist for up to five months even after treatment [4].

The foot lesions lead to disorders of the locomotor system, associated with pain, tissue damage, and impaired homeostasis, and can induce a systemic acute phase response (APR) [5]. APR is a systemic inflammatory reaction triggered by the body following tissue damage [6,7] and manifests itself through the action of pro-inflammatory cytokines, such as interleukin-6 (IL-6), and acute phase proteins (APPs), which limit microbial growth and contribute to maintaining the organism’s balance. During this response, APPs protect the humoral and cell-mediated immune systems from unnecessary host damage [8]. APR is nonspecific with respect to the cause and occurs regardless of the etiology, which can be infectious, traumatic, immunological, neoplastic, or other. Acute phase proteins are classified as positive or negative based on the changes in their concentration during the response. Positive APPs, such as haptoglobin (Hp), serum amyloid A (SAA), C-reactive protein (CRP), and fibrinogen (Fb), show a significant increase, while negative APPs, such as albumin and transferrin, decrease [6,7,8]. Positive APPs can be further subdivided into major and moderate based on their response to pro-inflammatory stimuli [6,7,8]. The major proteins, such as CRP and SAA, show a rapid increase and decrease in serum concentration, with increases that can reach up to 100 times the baseline level. Moderate proteins, including Hp, α-1-acid glycoprotein (AGP), and ceruloplasmin (Cp), show a more modest increase, ranging from 1 to 10 times the baseline level, and reach their peak and serum reduction more slowly [9]. In serum protein electrophoresis, APPs preferentially migrate into the α_1_ and α_2_ fractions (fibrinogen, Hp, CRP, SAA), increasing α-fractions in acute inflammation. Some minor immunoglobulins and APPs are localized in the β-fraction, while widespread increases in γ-fractions indicate polyclonal gammopathies, typical of chronic inflammation, prolonged infections, or neoplasia. In parallel, albumin and transferrin, being APP-negative, undergo a reduction, resulting in a lowering of the albumin-to-globulin (A/G) ratio, typical of acute or chronic inflammatory responses [10]. In bovine species, elevated levels of APPs, such as SAA and Hp, have been detected in the blood of animals affected by various pathologies, including lower respiratory diseases, mastitis, and hoof disorders [8]. In healthy animals, however, these proteins are absent or present in negligible amounts [1]. Therefore, assessing acute phase proteins represents a useful indicator of the systemic impact of foot pathologies in ruminants, offering a potential tool for their early diagnosis and monitoring of inflammatory status. Since local temperature increases in the presence of inflammation, the use of infrared thermography in the diagnosis of foot diseases can be an effective and useful tool [11]. The surface temperature of the skin and extremities depends primarily on blood perfusion and tissue metabolism but can also be influenced by individual differences between animals and environmental factors [11,12,13]. In healthy cattle, for example, the hind feet are generally warmer than the forefeet, with the posterior lateral claw being warmer, while no significant differences between the claws are observed in the forefeet [11]. It has been observed that, as the severity of the infection increases, the maximum hoof temperature does not necessarily increase. Rather, thermal energy tends to spread more uniformly, resulting in an increase in the average temperature of the entire foot surface [12]. Modern thermal imaging cameras used on farms are able to measure changes in skin temperature with an accuracy of approximately ±0.1 °C, allowing thermal anomalies to be identified before they are palpable. This is particularly important for the early diagnosis of foot lesions, when the animal does not yet exhibit pain or changes in foot strike [11], which then allows the animals to undergo a clinical examination or exploratory trimming [13].

In view of the above considerations, the current study aimed to evaluate the clinical progression of foot lesions in lactating dairy cows by combining clinical examination with hematochemical analyses and infrared thermography, in order to assess the effectiveness of therapeutic interventions and the evolution of inflammation over time.

## 2. Materials and Methods

### 2.1. Study Design and Animals

All treatments, housing, and animal care described below were conducted in accordance with the standards recommended by Directive 2010/63/EU of the European Parliament and of the Council on the protection of animals used for scientific purposes. The Ethics Statement was approved by the Animal Care and Use Committee of the University of Padua (protocol n. 103549/2024).

The study was conducted on an intensive free-stall dairy farm housing 100 Italian Friesian cows. Animals were grouped according to production stage (lactating, dry, and heifers), housed in dedicated sheds, and fed a total mixed ration consisting of hay and concentrates. The farm was equipped with individual cubicles, and the feeding and walking alleys, as well as the milking parlor, had solid concrete flooring. The floor was constructed of reinforced concrete strengthened with welded steel mesh to withstand and evenly distribute operational loads and ground counterthrusts. While still fresh, the concrete surface was tamped to create a hexagonal pattern. The resulting grooves were approximately 8 mm wide and 7 mm deep, improving traction and reducing the risk of slipping. At the time of the study, the flooring had been in use for more than five years. Floors were cleaned regularly using automatic scrapers. All animals were free from internal and external parasites. Body Condition Score (BCS; 0–5 scale) was recorded for each cow prior to the study. Cows were fed a balanced diet formulated to meet the nutritional requirements for mid-lactation (Table 1). Water was available ad libitum.

The study included 10 lactating cows (mild-lactation; 2–3 years old; mean body weight 625 ± 115 kg; BCS 2.2 ± 0.3) affected by hoof diseases (DG), and 5 healthy lactating cows (mild-lactation; 2–3 years old; mean body weight 612 ± 102 kg; BCS 2.4 ± 0.3) selected as the control group (CG).

All cows had previously participated in a routine maintenance claw-trimming program, receiving hoof trimming twice yearly (approximately 70 ± 15 days before and 50 ± 15 days after calving). Scheduled sessions were performed by a professional veterinarian-claw trimmer about two months before calving. During trimming, cows were placed in an upright Dutch hoof crate equipped with a headlock and a manually operated rope foot lift. Each limb was raised individually using a rope secured around the mid-diaphysis of the third metatarsal bone. All feet were thoroughly cleaned with cold water and dried before trimming to remove stained or overgrown hoof horn tissue. Only one leg was lifted at a time to minimize stress and ensure animal welfare. Lame cows were examined and their condition was recorded during each session. For the study, selected cows were guided into the chute, where affected feet were carefully examined. A full clinical assessment was performed, with particular attention to the cow’s reaction to palpation and pain sensitivity. In cases where both feet were affected, the foot exhibiting the most severe clinical signs was selected. The same procedure was applied to healthy cows. In addition to dynamic gait scoring, a static lameness score was also assessed. For the purposes of the study, selected cows were guided into the trimming chute, where affected claws were carefully examined. A comprehensive clinical examination was performed, with particular attention to pain response upon palpation and manual manipulation. In cases where lesions were present in more than one claw, the claw exhibiting the most severe clinical signs was selected for classification. The same examination protocol was applied to clinically healthy cows to ensure consistency of assessment.

In addition, herd-level lameness prevalence was evaluated using both dynamic (gait scoring) and static lameness scoring methods, in accordance with established animal-based assessment protocols.

Dynamic gait scoring was applied to lactating cows, dry cows, and pregnant heifers. Each animal was individually observed while walking in a straight line on a hard, level, non-slip surface under normal farm conditions. Cows were assessed from the side and/or from behind and were not evaluated while turning. Lameness was defined as an abnormality of movement characterized by alterations in step timing, temporal rhythm, and weight-bearing distribution among the four limbs. Animals were classified at the individual level as follows: Score 0 (not lame): regular timing of steps and equal weight-bearing on all four limbs; Score 1 (moderately lame): imperfect temporal rhythm resulting in an uneven gait or limp; Score 2 (severely lame): marked reluctance to bear weight on one limb or involvement of more than one limb. In addition to dynamic assessment, a static lameness score was also recorded. Because locomotion scoring may not always be feasible, static evaluation was based on validated behavioral and postural indicators, including preferential resting of one foot, standing on the edge of a step to reduce weight-bearing, frequent weight shifting between limbs (“stepping”), repeated lifting of the same foot, and reluctance to bear weight during movement. Each cow was first observed while undisturbed, then gently encouraged to shift weight laterally, and finally reassessed after returning to a normal standing posture. If the cow had been lying down, assessment was postponed for 3–4 min after rising. Cows showing none of the listed indicators were classified as score 0 (not lame), whereas cows showing at least one indicator were classified as score 2 (lame). At herd level, the percentage of cows in each static score category was calculated [14]. The animals were evaluated and sampled at three different time points: clinical diagnosis of the foot pathology (T0), recording of physiological parameters, blood sampling and acquisition of thermographic images; 24 h after treatment by functional or therapeutic trimming (T1), including clinical examination, measurement of physiological parameters, blood sampling and acquisition of thermographic images; 10 days after treatment (T2), including the same surveys to evaluate the clinical and laboratory evolution of the pathology.

### 2.2. Blood Collection and Analysis

Blood was collected by jugular venipuncture using two types of Vacutainer tubes: one containing ethylenediaminetetraacetic acid (EDTA) (Terumo Co., Tokyo, Japan) for hematology analysis and one containing clotting activators (Terumo Co., Tokyo, Japan) for serum separation. The EDTA samples were used to determine red blood cells (RBCs), white blood cells (WBCs), platelets (Plts), hemoglobin (Hb), hematocrit (Hct), mean corpuscular volume (MCV), mean corpuscular hemoglobin (MCH), and mean corpuscular hemoglobin concentration (MCHC). Whole blood glucose (GLU) was also determined using a portable meter (Accutrend Plus^®^, Roche Diagnostics, Rotkreuz, Switzerland) immediately after collection. Samples were kept refrigerated and analyzed within 24 h of collection. Hematological analyses were performed using a HeCo Vet C automated cell counter (SEAC, Florence, Italy). For differential leukocyte counting, a peripheral blood smear was prepared from each sample, air-dried, and then stained with the Dif-Stain kit (Titolchimica srl, Rome, Italy). Microscopic observation was performed with a Nikon Eclipse e200 optical microscope (Nikon Instruments Europe BV, Amsterdam, The Netherlands), manually counting 100 cells for each sample. The differential leukocyte count was calculated based on the percentages obtained from this microscopic evaluation. Serum was obtained from blood samples collected in tubes containing clotting activators after centrifugation at 4000 rpm for 15 min. The samples were then divided into aliquots and stored at –20 °C until analysis. Serum total protein (TP) concentration was determined using the Biuret method via a UV spectrophotometer (SEAC, Florence, Italy), and protein fractions were determined using an automated system (SELVET 24, Seleo Engineering, Naples, Italy) according to the manufacturer’s instructions. For each sample, 25 µL of serum was loaded into numbered wells; each sampling unit allowed the insertion of up to 24 positions, divided into three supports of eight samples. Electrophoresis was conducted on cellulose acetate membranes for 28 min at a voltage of 450 V. At the end, the membranes were automatically fixed, then stained with Ponceau Red S (acidic pH) for 10 min and left to dry at 37 °C. After approximately 15 min, during which the acetic acid decolorization and final drying phases were completed, the membranes were subjected to densitometric readings to quantify the protein bands. The staining intensity was directly proportional to the amount of protein present. From the densitometric pattern obtained, the individual fractions were represented by peaks of varying width and height, the area of which corresponded to the percentage of protein in each fraction. Electrophoretic separation allowed the main protein components to be highlighted in accordance with the manufacturer’s specifications: starting from the anodic (positive) pole, the most pronounced albumin peak was observed, followed by the α-fraction peak, then the β1- and β2-fraction peaks, and finally, near the cathodic pole, the γ-fraction, characterized by a broader and less pronounced curve.

### 2.3. Infrared Thermography and Thermographic Analysis

Thermal images of the limbs were acquired for each animal using a digital infrared thermal imaging camera (ThermaCam P25, model EL, FLIR Systems, Boston, MA, USA).

Before each acquisition, the paws were thoroughly cleaned and trimmed to remove any dirt or organic material, thus avoiding distortion or artifacts in the thermal recording. After washing and drying, a waiting period of approximately five minutes was allowed to allow peripheral blood flow to return to pre-wash physiological conditions. Thermal images were taken indoors, with the animals immobilized in the stable in an upright position, to minimize the influence of external factors on the measurements. The average ambient temperature recorded during the various acquisition times in cows was 19.7 ± 4.2 °C with a relative humidity (RH) of 62.5%.

All images were obtained by maintaining a fixed distance of 0.7 m between the thermal imaging camera and the subject’s skin surface, minimizing any environmental variations. The thermal imaging camera’s acquisition parameters were as follows: a temperature range of 10 to 40 °C; skin emissivity set to 0.98; a reflected air temperature (Trifl) of 20 °C; a distance (Dist) of 0.7 m; and a field of view (FOV) of 23°.

The detector consisted of an uncooled microbolometer with a focal plane array (FPA), featuring a resolution of 320 × 240 pixels, a thermal sensitivity of 0.08 °C at 30 °C, a spatial resolution (IFOV) of 1.3 mrad, a spectral range of 7.5 to 13 µm, and an accuracy of ±2 °C.

Automatic corrections were also applied based on the parameters entered by the operator (reflected ambient temperature, distance, relative humidity, and atmospheric transmission). Temperature measurements were performed in four specific areas of the hindfoot: the central zone (R1), the interdigital area (R2), and the regions corresponding to the lateral (R3) and medial (R4) claw. For each of these regions of interest, the mean absolute temperature was determined using dedicated thermographic analysis software (Thermacam Researcher Basic 2.8, FLIR, Wilsonville, OR, USA).

### 2.4. Foot Treatment

Therapeutic hoof trimming procedures in cows were performed by a trained veterinarian. The animals were placed in a Dutch hoof crate equipped with a safety lock and a hydraulic rope-based hoof lift system. Each limb was lifted using a rope attached to the midshaft of the third metatarsal. The feet were thoroughly cleaned to remove altered or excess horny tissue, following the Dutch Technique [15]. During hoof trimming, only one limb was lifted at a time to ensure maximum comfort and reduce stress. Subsequently, a complete clinical examination was conducted, evaluating the cow’s response to palpation, and the pathological condition of the hoof was confirmed. In some animals with severe hoof damage, an orthopedic insole was applied to promote proper healing.

### 2.5. Statistical Analysis

The collected data were preliminarily tested for normal distribution using the Kolmogorov–Smirnov test. Since the data distribution was found to be normal (*p* > 0.05), statistical analysis was subsequently conducted. To evaluate the effect of foot pathologies and of time on the concentration of investigated hematochemical and to assess significant temperature variations between different anatomical areas of the foot and, a two-way analysis of variance (ANOVA) for repeated measures was applied. In cases where statistically significant differences emerged, a Bonferroni post hoc test was performed for multiple comparisons of the groups. All statistical analyses were performed using STATISTICA 7 software, version 7.0 (StatSoft Inc., Tulsa, OK, USA).

## 3. Results

**Figure 1:**
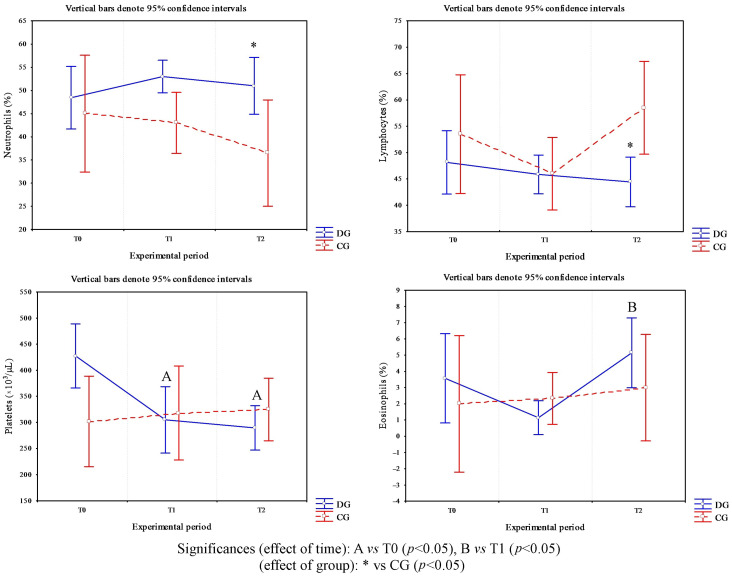
Trend of the values of platelets, lymphocytes, neutrophils and eosinophils measured in healthy cows (CG) and in cows with foot diseases (DG) during the experimental period (clinical diagnosis of the foot pathology, T0; 24 h after treatment, T1; 10 days after treatment, T2). The symbol * indicates difference between groups; the letters A and B indicate the difference among time points.

**Figure 2:**
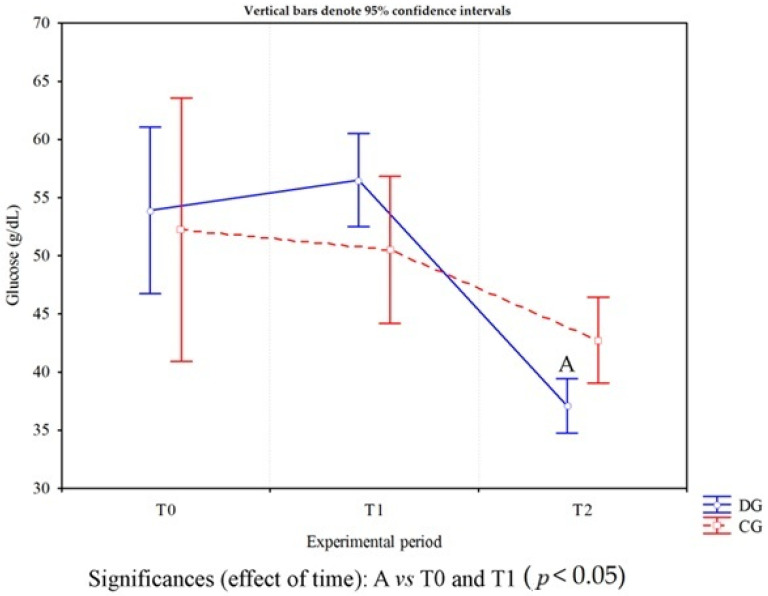
Trend of blood glucose values measured in healthy cows (CG) and in cows with foot diseases (DG) during the experimental period (clinical diagnosis of the foot pathology, T0; 24 h after treatment, T1; 10 days after treatment, T2). The letter A indicates the difference among time points.

**Figure 3:**
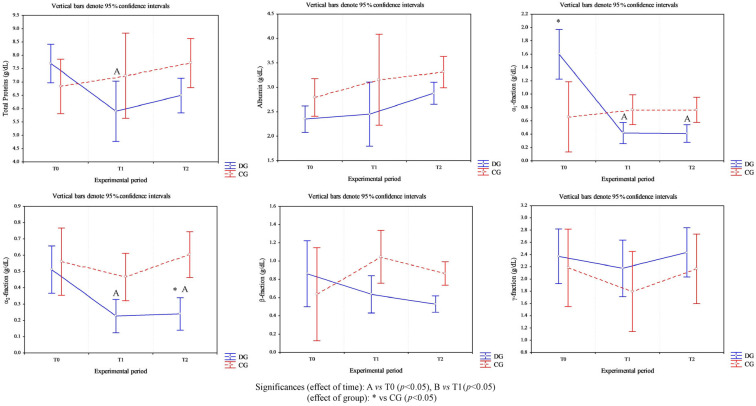
Trend of the values of serum total proteins and their fractions (i.e., albumin, α1-, α2-, β- and γ-fraction) measured in healthy cows (CG) and in cows with foot diseases (DG) during the experimental period (clinical diagnosis of the foot pathology, T0; 24 h after treatment, T1; 10 days after treatment, T2). The symbol * indicates difference between groups; the letters A and B indicate the difference among time points.

**Figure 4:**
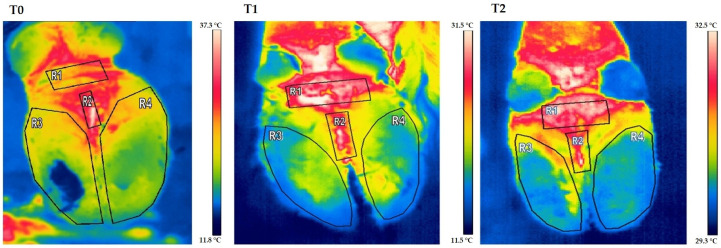
Representative thermographic image of foot temperature in the four specific areas of the hindfoot of a diseased cow: the central zone (R1), the interdigital area (R2), and the regions corresponding to the lateral (R3) and medial (R4), recorded during the experimental period (clinical diagnosis of the foot pathology, T0; 24 h after treatment, T1; 10 days after treatment, T2).

**Figure 5:**
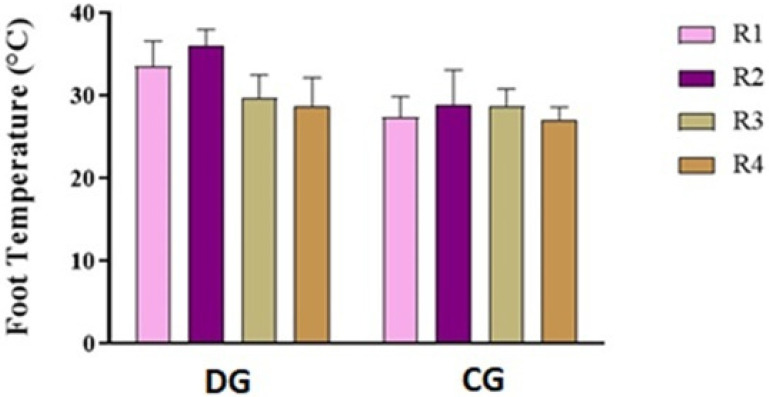
Foot temperature values recorded in areas R1, R2, R3 and R4 in diseased (DG) and healthy (CG) cows during the clinical diagnosis of the foot pathology (T0).

**Figure 6:**
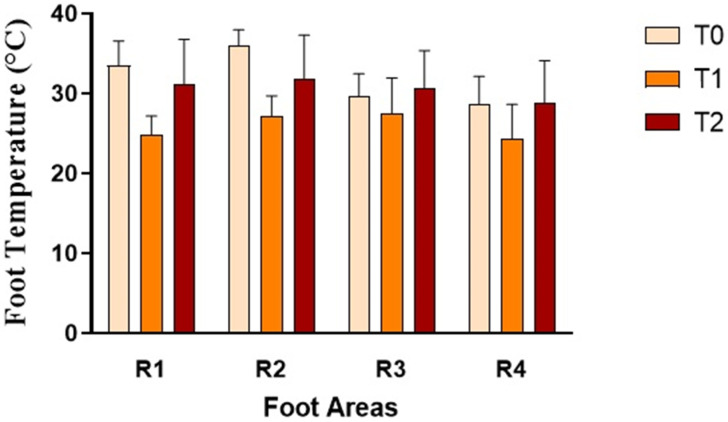
Foot temperature values recorded in areas R1, R2, R3 and R4 in diseased cows (DG) during the experimental period (clinical diagnosis of the foot pathology, T0; 24 h after treatment, T1; 10 days after treatment, T2).

Cows affected by foot diseases at T0 presented with various pathologies, including ulcers, interdigital dermatitis, digital dermatitis, laminitis, and white line disease, with variable localization between the forelimbs and hindlimbs. A detailed summary of the lesions found in cows is shown in Table 2. Statistical analysis of parameters in cows highlighted a significant effect of the group on the following parameters: neutrophils (*p* < 0.05) and lymphocytes (*p* = 0.03) (Figure 1). Furthermore, an effect of time was highlighted on PLTs (*p* < 0.05), eosinophil values (*p* < 0.005) (Figure 1) and glucose concentrations (*p* < 0.05, Figure 2) in DG. Specifically, PLT values were higher at T0 than T1 and T2, eosinophils values were higher at T2 than T1, and glucose concentration was lower at T2 than T0 and T1. Statistical analysis of protein fractions in cows highlighted a significant effect of time in the DG on the following parameters: TP (*p* < 0.05), with higher values at T0 than T1, α1- (*p* < 0.05) and α2-fractions (*p* < 0.05), with higher values at T0 than T1 and T2. A significant group effect was found in α1- (*p* < 0.05) and α2-fractions (*p* < 0.05) with higher values of both parameters in diseased subjects compared to healthy ones at T0 (Figure 3). In Figure 4, a representative thermographic image of foot temperature in the R1-4 regions of a diseased cow recorded at T0, T1 and T2 was reported. Animals with lesions showed higher temperatures than healthy subjects (*p* < 0.001), especially at T0 (Figure 5) and in particular animals affected by hoof diseases showed lower foot temperature values at T1 compared to T0 and T2 (*p* < 0.001) in the R1-R4 regions. A statistically significant effect of sampling time was observed on foot temperatures detected in the various regions of the foot (R1, R2, R3 and R4). In particular, thermography highlighted significantly higher thermal values (*p* < 0.01) in the central (R1) and interdigital (R2) areas of the hoof compared to the lateral (R3) and medial (R4) areas (Figure 6).

## 4. Discussion

Lameness in ruminants represents a major global health concern, significantly impacting both animal welfare and economic losses resulting from declining production and increased healthcare and culling costs [4,13]. Despite advances in herd management, hoof disorders remain a major challenge for the sustainability of livestock farms. This study aimed to evaluate the clinical progression of hoof disorders following treatment, monitoring changes in both thermographic patterns and blood chemistry parameters. To achieve this, the functional trimming technique (Dutch five-step method), one of the most standardized approaches for correcting hoof disorders [16], was applied to dairy cows. In animals with severe lesions, such as sole ulcers, treatment was complemented with the application of a therapeutic insole to relieve the weight on the affected limb.

The use of the Dutch method in this study allowed for the assessment of its impact on lameness, highlighting its central role in the management of hoof disorders in dairy cows. Numerous authors have already documented its effectiveness in both prevention and treatment [12,16,17]. The method aims to restore correct claw biomechanics by leveling the abaxial and axial walls, making them perpendicular to the metatarsals, and by more uniformly distributing the load between the claws [12]. This helps contain excessive horn growth, uniform sole thickness, and remove necrotic tissue, creating a more oxygenated microenvironment that limits the proliferation of anaerobic microorganisms [4] and results in a reduction in pain and improved gait [16]. Clinical and experimental studies confirm that cows treated with trimming have a lower incidence of lameness and a lower likelihood of developing hoof lesions compared to untreated cows [17] and that corrective trimming significantly reduces the percentage of lame cows and improves locomotion scores within weeks following surgery [3,17].

The rapid recovery observed at T1 is consistent with the findings of Laiju et al. [18] who found that therapeutic trimming, often combined with dressings and bandages, promotes relatively rapid healing of painful sole lesions, with coverage of the horny defect within 20 days. The reappearance of clinical signs at T2, however, highlights the limitations of trimming if not incorporated into a broader management strategy. Orthopedic correction alone, in fact, is not sufficient to prevent and permanently control lameness, particularly in infectious forms (e.g., digital dermatitis), unless accompanied by adequate environmental management [3,16,19]. For example, it has been shown that recovery rates are more favorable in tie-stall barns than in free-stall barns, where constant exposure to dirty floors or slurry hinders recovery [17]. In this context, even dressings and bandages, while appropriate in conditions such as hoof bleeding, can become an additional risk factor: in unhygienic environments, the animal’s passage over contaminated surfaces (feces, slurry) facilitates bandage contamination, potentially prolonging healing times and leading to irritation or complications, such as those highlighted at T2 [4]. Recent meta-analyses confirm that lack of access to pasture, concrete floors, and inadequate lying surfaces are the main risk factors for lameness and that a reduction in prevalence can only be achieved through improved stable structure and management [19]. It should also be remembered that the trimming procedure itself is a stressful stimulus, associated in the short term with decreased production, reduced walking, and increased resting time [3,17]. The findings obtained at T0, T1, and T2 using the Dutch method are supported by the trend observed for complete blood counts, biochemical parameters, thermographic images, and serum protein fractions. The eosinophil count showed significantly higher values in lame animals at T0 compared to controls, with a subsequent slight post-treatment reduction and a renewed increase 10 days after trimming. This trend suggests an initial favorable response to treatment, followed by a recurrence that could be likely related to the trimming procedure which is likely known to be itself a stressful stimulus. The increase in eosinophils in affected animals at T0 and T2 is consistent with that reported in animals with hoof disease [20,21]. From a pathophysiological perspective, this increase is often associated with the release of histamine, which in inflammatory processes, such as laminitis, acts as a potent chemotactic agent for these cells [20]. Moreover, the higher neutrophils values in lame cows compared to controls is consistent with an acute inflammatory response related to hoof disease and consistent with data reported in cattle with ulcers or other hoof lesions [22]. A further increase was observed 24 h post-trimming (T1), which can be interpreted as a response to the acute insult represented by the trimming procedure acting as an additional inflammatory stimulus; moreover, despite a clinical flare-up, a slight decrease in neutrophil values was recorded at T2 compared to T1, indicative of a partial response to the elimination of the primary inflammatory cause. A complete recovery would likely have resulted in a more marked reduction in the neutrophil:lymphocyte ratio, as described by Laiju et al. [18] after therapeutic hoof trimming. The maintenance of relatively high neutrophil values at T2, together with a still increased neutrophil/lymphocyte ratio, indicates that the inflammatory process has not completely resolved. The lymphocyte profile was mirrored: the lame group showed persistent lymphopenia at T0, T1, and T2 compared to the controls. In ruminants, during the acute phase response, adrenal activation can lead to the destruction or sequestration of lymphocytes, resulting in a reduction in their circulating number [23]. Although there were no significant differences between neutrophils and lymphocytes in a specific study on laminitis [20], the tendency towards lymphopenia found in the present work is consistent with what was observed by Barbosa et al. [23], who reported significantly lower lymphocyte values in lame cows compared to healthy cows, in a typical stress response scenario. The failure of lymphocyte levels to rise to T1 and T2 confirms the persistence of an inflammatory and stress-related state, presumably linked to the failure to adapt management, which prevents the complete recovery of the hematological profile. Overall, the combination of neutrophilia, lymphopenia, and an increased neutrophil/lymphocyte ratio is characteristic of animals subjected to chronic stressful conditions, such as incompletely resolved lameness and unfavorable environmental conditions [22]. Regarding platelets, the diseased group showed significant thrombocytosis compared to controls at T0. This increase is consistent with evidence indicating higher platelet counts in cattle with locomotor disorders [21,23], likely related to the stress and pain associated with maintaining a balanced posture and walking [24]. Reactive or secondary thrombocytosis is in fact described as a consequence of cytokine release and is closely associated with inflammation and stress [25,26,27]. Platelets, in addition to their role in hemostasis, participate in the inflammatory response through the release of vasoactive and chemotactic mediators. In the time points following treatment (T1 and T2), a progressive decrease in platelet count was observed. This trend differs from that documented in other studies, in which a further increase in platelets was observed during the convalescence phase. Gimranov et al. [21] described an increase in the number of platelets in cows with purulent–necrotic processes 10 days after treatment, which was interpreted as a sign of regenerative processes and acceleration of ongoing coagulation; similarly, Laiju et al. [18] associated an increase in platelet count within 20 days of trimming with functional recovery of the limb, improved posture and a reduction in pain. In the present study, on the contrary, the reduction observed at T1 and T2 can be interpreted as indicative of an incompletely initiated healing process: the cessation of the initial hyperacute stimuli (T0) was not followed by an effective regeneration phase, likely due to the failure to modify predisposing factors and/or due to the trimming procedure [18,21].

The glucose trend reflected the different phases of stress and metabolic adaptation. At T0, lame cows tended to have higher glucose levels than controls, although without statistical significance, consistent with the fact that one of the typical responses to stress is hyperglycemia, due to the mobilization of hepatic glycogen during injury or sepsis [28] and glycogenolysis associated with an increase in catecholamines and glucocorticoids [29]. Higher glucose levels have also been described in animals with sole ulcers [22,30]. Ten days after trimming (T2), consistent with the findings of Sadiq et al. [17], glucose values decreased in cows with hoof disease. This finding can be interpreted as an expression of increased energy expenditure related to the greater expenditure required for locomotion under conditions of pain and for the attempt to recover limb function. Lameness, in fact, alters posture and movement dynamics, forcing the animal to expend greater energy; the drop in blood glucose may therefore reflect this increased requirement [24], especially in the post-treatment adaptation phase.

The thermographic images obtained are consistent with both the clinical picture and blood chemistry parameters, confirming the usefulness of infrared thermography (IRT) as a diagnostic tool and for monitoring the progression of foot pathologies. When lameness is caused by inflammatory processes, local hyperemia translates into a true “thermal signature” detectable on the surface [11]. Foot lesions cause an increase in skin temperature, which is readily detectable by IRT [31,32]; current equipment allows for the detection of temperature differences in the order of ±1.5 °C, allowing for the early detection of variations not yet appreciable by palpation [11]. The significant temperature difference between healthy and lame animals at T0 observed in the study is therefore perfectly compatible with active local hyperemia. Consistent with the literature, the highest temperatures were recorded in the R1 (central) and R2 (interdigital) regions, areas characterized by greater vascularization and metabolic activity, as well as a lower degree of keratinization compared to the sole, which tends to present lower values [31,32]. These same regions are considered particularly sensitive for the detection of lesions such as digital and interdigital dermatitis [31], confirming the ability of IRT to localize the inflammatory focus [33]. Of clinical significance is the decrease in temperature recorded 24 h post-treatment in the limbs of lame subjects, compared to T0 values. This demonstrates how IRT can be used not only for diagnosis but also for assessing post-treatment progress. Wood et al. [34] documented a reduction in foot temperature six weeks after treatment, correlated with a decrease in inflammation; Alsaaod and Buscher [35] report that functional trimming can modify thermal values due to the different distribution of the load on the foot. However, it remains necessary to further investigate the thermographic response in the phase immediately following trimming, since the surface temperature can be affected both by the redistribution of the load and by the extent of pre-existing lesions. To obtain reliable measurements, IRT requires standardized acquisition protocols: controlled ambient temperature, adequate cleaning and drying of the hoof, and limitation of confounding factors such as direct sunlight and drafts [31,32,35]. The increase in thermal values recorded at T2 in the limbs of lame cows indicates a reactivation of the inflammatory process, consistent with blood chemistry and clinical data. IRT therefore appears to be a useful early indicator of relapse. This evidence draws attention to animal welfare issues on farms and the need for structural and management interventions [33]. Serum protein electrophoresis measured at T0 showed higher total protein, and α-1 and α-2 fraction values in diseased cows compared to healthy ones. This profile is compatible with what has been described in clinically lame cows, characterized by higher total protein concentrations, mainly due to the increase in α- and γ-fractions and the simultaneous reduction in albumin [22]. As a matter of fact, α fractions include several acute phase proteins (APPs), including haptoglobin (Hp) [22], which are considered sensitive biomarkers of the activation of the systemic inflammatory response in hoof pathologies [1,36]. Increased Hp is also described in the mildest clinical forms of lameness [22]. The combined increase in α1- and α2-fractions at T0, sites of electrophoretic migration of Hp and SAA, could reflect a systemic activation of the acute phase [22]. In parallel, a trend, albeit non-significant, toward lower levels of albumin, a negative acute phase protein that tends to decrease during inflammation, has been observed in affected cows. Dynamic changes observed in the values of protein fractions 24 h (T1) and 10 days (T2) after treatment allowed us to correlate their trend with clinical evolution. At T1, in conjunction with clinical improvement, a decrease in the values of α-fractions was observed suggesting a reduction in the acute inflammatory process. This decrease in the first 24 h is consistent with what was reported by Jawor et al. [37], according to which the reduction in the APPs in the post-treatment period demonstrates good therapeutic efficacy; similarly, other studies highlight a progressive normalization of the fractions as healing progresses [22]. However, the α1- and α2-fractions levels failed to fully normalize at T2 coinciding with the clinical reappearance of lameness signs at 10 days and suggesting the persistence of an inflammatory state. It is likely that this lack of improvement could be linked to the lack of corrective interventions in farm management (housing conditions, flooring), given that the environment is recognized as a determining etiological factor in the genesis and persistence of hoof pathologies [36,37]. The persistence of higher α-globulin values at T2 may, therefore, reflect a flare-up or a complication related to the recovery environment. Overall, the assessment of serum protein fractions provided objective support for the diagnosis and monitoring of the systemic inflammatory response associated with lameness. Serial determination at different time points could be suggested as a complementary tool to the clinical examination, useful for quantifying the severity of the pathology at onset, the short-term efficacy of the treatment and the impact of environmental factors in the recovery phase [37]. The results gathered in the current study highlight the importance of a multidisciplinary approach for diagnosis, treatment and monitoring of foot disorders in dairy cows, and support the use of thermography and serum protein analysis as complementary tools to assess the effectiveness of therapeutic protocols and to improve animal welfare and farm sustainability. However, the small simple size and the short surveillance period represent the main limitations of the study which call for further investigations on a larger population of animals affected by foot pathologies for longer observation periods in order to better and more thoroughly evaluate the course of the pathologies. Furthermore, comparative thermography and biochemistry analysis between cows with hoof abscesses and cow with laminitis would be worthy of investigation.

## 5. Conclusions

The results of this study confirm that footpad disorders are a major health problem in dairy ruminants, with significant repercussions on animal welfare and farm productivity. The approach used, which integrates clinical assessment, blood biochemistry analysis, and infrared thermography, allowed the progression of lesions to be monitored over time, providing a comprehensive overview of the response to treatment. In cows, functional trimming according to the Dutch method produced a significant clinical improvement, confirmed by the trend in inflammatory indices and acute phase proteins. The improvement observed at T1 and the subsequent flare-up at T2 confirm what has been reported in the literature: trimming is effective, but alone is not sufficient if the housing environment remains unfavorable. Infrared thermography has confirmed its usefulness as an early diagnostic tool and for monitoring clinical outcome. The observed thermal profile, with elevated temperatures in the R1 and R2 regions at T0 and a subsequent reduction after treatment, was consistent with inflammation-induced hyperemia. IRT has also proven useful for early identification of recurrences, as evidenced in T2 cows. The serial approach (T0–T1–T2) allows for objective detection of both improvement and any relapses, overcoming the limitations of clinical observation alone.

Some limitations, especially the small number of enrolled animals (*n* = 10 diseased cows; *n* = 5 control cows), the relatively short observation period, and the presence of comorbid conditions, limit the possibility of extending the results to the entire population. However, the findings have concrete practical value. In cattle, functional trimming must be integrated into a broader environmental management program—adequate flooring, hygiene, bedding, and pasture access—to ensure effective prevention and reduction in lameness. This pilot study confirms that a multidisciplinary approach combining functional trimming, IRT, and hematochemical monitoring effectively tracks the progression of foot lesions in dairy cows. Recovery is influenced by both treatment and environmental management, emphasizing the need for integrated herd management practices to improve animal welfare and farm sustainability.

## Figures and Tables

**Table 1:** Mean chemical composition (% of DM) of diets of dairy cows.

Parameter	Estimated Value
Crude Protein	16.8%
NDF	33.5%
ADF	20.5%
Starch	25.0%
NFC	38%
Ether Extract	5.8%
Calcium	0.95%
Phosphorus	0.43%
Magnesium	0.32%
Sodium	0.50–0.60%

**Table 2:** Location and typology of foot lesions in lame dairy cows.

	Cows with Foot Diseases	
**N**	Type of foot injury	Location
**1**	Interdigital dermatitis with abscess	Left hind foot
**2**	Digital dermatitis	Left hind foot
**3**	Laminitis, sole ulcer, digital dermatitis	Left and right hind foot
**4**	Digital dermatitis with abscess	Right hind foot
**5**	Digital dermatitis	Right front foot
**6**	Digital dermatitis	Left hind foot
**7**	White line disease	Right hind foot
**8**	Early laminitis	Right hind foot
**9**	Presence of a foreign body	Left hind foot
**10**	White line disease	Right hind foot

## Data Availability

Data is contained within the article.
